# Towards the fluorogenic detection of peroxide explosives through host–guest chemistry

**DOI:** 10.1098/rsos.171787

**Published:** 2018-04-11

**Authors:** Estefanía Almenar, Ana M. Costero, Pablo Gaviña, Salvador Gil, Margarita Parra

**Affiliations:** 1 Instituto Interuniversitario de Investigación de Reconocimiento Molecular y Desarrollo Tecnológico (IDM), Universitat de València-Universitat Politècnica de València, Valencia, Spain; 2 Departamento de Química Orgánica, Universitat de València, Doctor Moliner 50, 46100, Burjassot, Valencia, Spain; 3 CIBER de Bioingeniería Biomateriales y Nanomedicina (CIBER-BBN), Spain

**Keywords:** peroxide explosives, fluorescent sensors, cyclodextrins, host–guest chemistry

## Abstract

Two dansyl-modified β-cyclodextrin derivatives (**1** and **2**) have been synthesized as host–guest sensory systems for the direct fluorescent detection of the peroxide explosives diacetone diperoxide (DADP) and triacetone triperoxide (TATP) in aqueous media. The sensing is based on the displacement of the dansyl moiety from the cavity of the cyclodextrin by the peroxide guest resulting in a decrease of the intensity of the fluorescence of the dye. Both systems showed similar fluorescent responses and were more sensitive towards TATP than DADP.

## 1. Introduction

The development of rapid and sensitive detection methods for homemade explosives has become an urgent need for obvious reasons. Of particular interest in this regard is the group of peroxide explosives, such as triacetone triperoxide (TATP) or diacetone diperoxide (DADP). Although TATP has an explosive power close to that of TNT, it has not been extensively used as a military explosive, due to its low chemical stability, high sensitivity to mechanical stress and high volatility [1–3]. However, due to its easy synthesis from readily available materials TATP has been used in several terrorist attacks in recent years, as in London, Paris or Brussels [4].

**Scheme 1.**
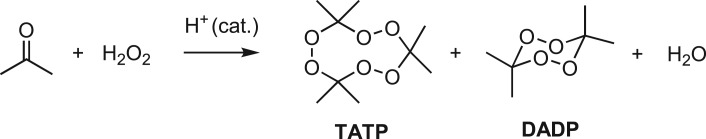
Synthesis of TATP.

TATP can be easily prepared from acetone and hydrogen peroxide in the presence of an acid catalyst such as HCl, HNO_3_ or H_2_SO_4_ (scheme 1). Under these conditions, TATP is always contaminated with small amounts of the dimeric compound DADP (*ca* 5–10% depending on the acid catalyst). TATP is the kinetic product while DADP is the thermodynamic one. In fact, in the presence of small traces of mineral acid, TATP can be slowly converted into DADP [1–3,5,6].

Peroxide explosives are difficult to detect by direct optical methods due to their lack of chromophores. TATP and DADP are non-fluorescent molecules with no absorption in the UV-vis. Thus, a variety of instrumental analytical methods have been developed for their detection, such as electrochemical methods [7], chromatographic and spectroscopic analysis [8,9], mass spectrometry [10] or immunoassays [11,12]. However, these detection strategies usually require expensive and non-portable instrumentation and complex sample pretreatment. These drawbacks can be overcome using optical chemosensors and probes, but their development is much less advanced and most of them are based on the detection of the acid decomposition products of the peroxide explosives, such as H_2_O_2_ or acetone [13,14]. In this sense, it is worth mentioning the colorimetric sensor arrays developed by Suslick's group [15–17].

**Figure 1.**
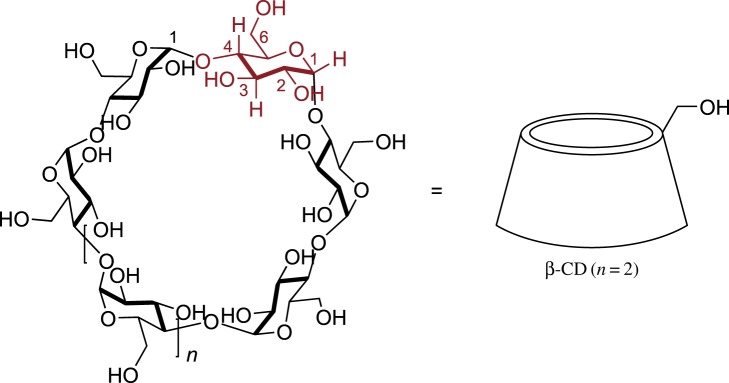
Structure of alpha-CD (*n* = 1), beta-CD (*n* = 2) and gamma-CD (*n* = 3).

We wanted to explore a new approach towards the direct fluorogenic detection of peroxide explosives such as TATP or DADP using host–guest sensory systems. TATP and DADP are small hydrophobic molecules, with sizes similar to those of the corresponding branched cycloalkanes [1]. Thus, as suitable hosts we decided to use cyclodextrins (CDs). CDs are cyclic oligosaccharides consisting of α-1,4-linked glucopyranose units. The most common CDs are α-, β- and γ-CDs (containing six, seven and eight glucose units respectively) (figure 1). Cyclodextrins are known to form inclusion complexes in aqueous solutions with a large variety of hydrophobic organic molecules of appropriate sizes.

**Figure 2.**
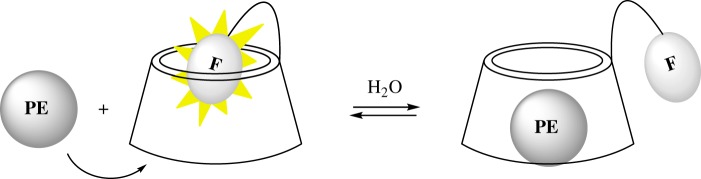
Paradigm of sensing mechanism using cyclodextrins derivatives with a dye connected through a flexible linker.PE: peroxide explosive, F: fluorophore.

The use of chromophore-modified CDs as fluorescent sensors for organic molecules has been extensively developed by Ueno and co-workers, dansyl being the most studied chromophore [18–21]. The sensing paradigm is depicted in figure 2. The optical properties of the dyes change upon encapsulation due to restricted rotations or changes in polarity of the microenvironment. In general, the fluorophore (e.g. the dansyl group) shows higher fluorescence when encapsulated inside the hydrophobic cavity of the cyclodextrin, forming a self-inclusion complex. Upon binding of a suitable competitive guest, the fluorophore is pushed out of the cavity and exposed to the bulk aqueous solution, resulting in a decrease of its fluorescence intensity (‘turn-off sensor’) [22].

Herein we report a new approach towards the direct optical detection of peroxide explosives (TATP or DADP) based on the use of new dansylamide-appended β-cyclodextrins as fluorescent host–guest sensors.

## 2. Results and discussion

**Figure 3.**
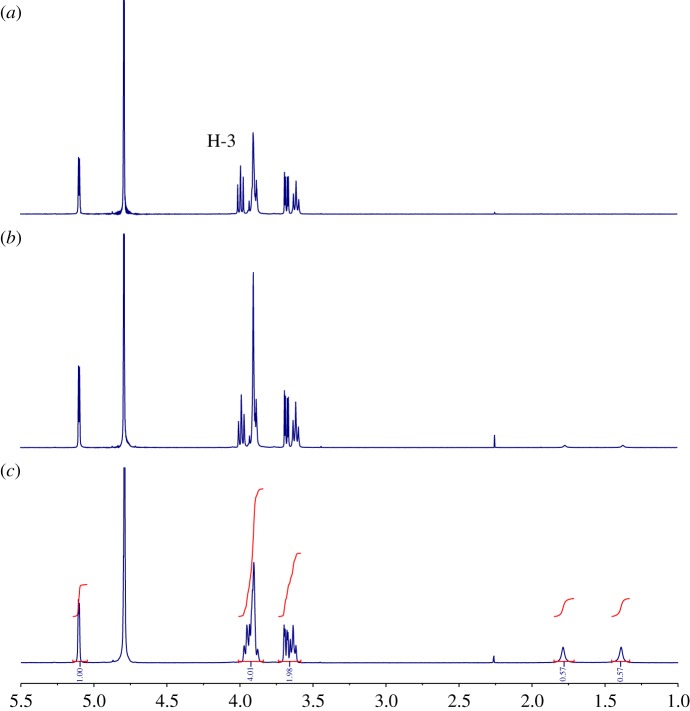
^1^H-NMR spectra (D_2_O, 500 MHz) of (*a*) β-CD; (*b*) β-CD + DADP and (*c*) β-CD + DADP sonicated for 2 min.

Prior to the synthesis of the dansylamide-appended β-cyclodextrins we decided to study by ^1^H-NMR the complexation properties of β-CD towards TATP and DADP. Thus, D_2_O solutions of β-CD were treated with an excess of solid TATP or DADP, shaken, filtered and their ^1^HNMR spectra were recorded. In the presence of TATP, very little changes in the signals of the cyclodextrin were observed. However, in the presence of DADP a small shift in the H-3 protons of β-CD to higher field was observed, and two new signals corresponding to the axial and equatorial methyl groups of DADP appeared in the spectrum (figure 3). It is worth mentioning that in the absence of cyclodextrin, no signals corresponding to DADP or TATP can be observed in the ^1^H-NMR spectra in D_2_O, which is consistent with the very low solubility of these peroxide explosives in water. Furthermore, when the mixture of β-CD and DADP was sonicated for 2 min before filtration, a highfield shift of H-3 of *ca* 0.04 ppm was observed together with an increase of the signals corresponding to DADP. Integration of the NMR signals led to a ratio β-CD/DADP of around 1.5. This result would suggest mixtures of 1 : 1 and 2 : 1 β-CD/DADP complexes.

**Scheme 2.**
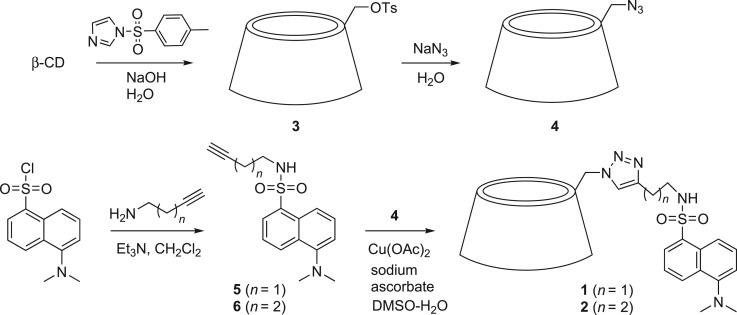
Structure and synthesis of compounds **1** and **2**.

In view of these results we decided to synthesize sensors **1** and **2** in order to evaluate their ability for sensing peroxide explosives. Sensors **1** and **2** consist of a β-cyclodextrin in which one C-6 of the primary face has been functionalized with a dansyl fluorophore through a flexible linker, using azide-alkyne click chemistry. The synthesis of these fluorophore-CD conjugates is shown in scheme 2.

First, regioselective monotosylation of one primary hydroxyl group in β-CD with 1-tosylimidazole under standard conditions [23] led to mono-6-tosyl-β-CD (**3**) which was then reacted with sodium azide to yield the mono-6-deoxy-6-azido derivative **4** [24]. On the other hand, reaction of dansyl chloride with the appropriate linear alkynamine led to terminal alkyne-functionalized dansyl derivatives **5** and **6**. Finally, the key step of the synthesis was the copper (I)-catalysed azide-alkyne cycloaddition between 6-azido-β-CD **4** and dansyl fluorophores **5** or **6**, which led to the corresponding dansyl-β-CD conjugates **1** and **2** through the formation of 4-alkyl-1,2,3-triazole linkers [25].

**Figure 4.**
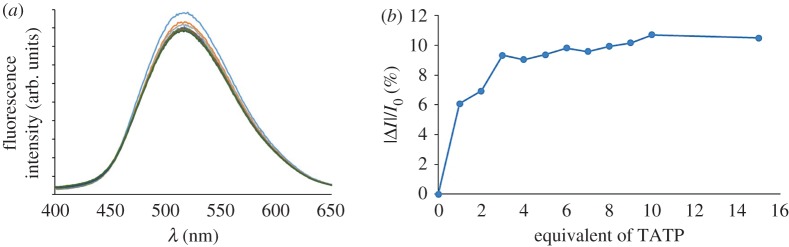
(*a*) Fluorescence emission spectra of **1** (1 × 10^−6^ M in H_2_O : MeOH 95 : 5, *λ*_exc_ = 340 nm) upon addition of increasing amounts (0–15 µM) of TATP. (*b*) Changes in the fluorescence intensity at *λ*_em_ = 518 nm of solutions of **1** (1 × 10^−6^ M in H_2_O : MeOH 95 : 5) in the presence of increasing amounts of TATP (*λ*_exc_ = 340 nm).

The spectroscopic properties of sensors **1** and **2** were evaluated in water : methanol (95 : 5) mixtures (1 × 10^−6^ M). Both compounds exhibit an absorption band around 340 nm and a fluorescence emission band at *λ*_em_ at 518 nm (*λ*_exc_ = 340 nm) as expected for a dansyl fluorophore [18–21] (see figure 4 for compound **1**), with a quantum yield of 0.18 (relative to fluorescein) [26]. Fluorescence titration experiments were performed with H_2_O : MeOH (95 : 5) solutions of compounds **1** or **2** (1 ×10^−6^ M) in the presence of increasing amounts of methanolic solutions of TATP or DADP. In preliminary experiments at room temperature, both compounds showed a moderate decrease in their fluorescence intensity (*ca* 3–6%) in the presence of the peroxide explosives, this effect being stronger for DADP than for TATP in both sensors (see electronic supplementary material). In order to improve the sensitivity or the detection, we repeated the experiments, heating the mixture of sensor and peroxide explosive at 40°C for 10 min before measuring its fluorescence spectra. Under these conditions, the sensitivity towards TATP could be significantly increased, and sensor **1** experienced a decrease in its fluorescence intensity of around 11% in the presence of excess of TATP and *ca* 7% in the presence of DADP (see figure 4 for TATP). It is noteworthy to mention that titration experiments with **1** in the presence of increasing amounts of adamantane (which forms effective inclusion complexes cyclodextrins) led to a similar decrease (10–11%) in its fluorescence intensity (see electronic supplementary material).

A possible explanation for this somehow modest sensitivity towards TATP and DADP could be their very low solubility in water solutions, which prevents reaching concentrations of the peroxide explosives high enough to observe a more significant displacement of the dansyl fluorophore by the explosive guest. However, titration experiments carried out with less polar solvent mixtures, such as MeOH, 10% H_2_O or MeCN, 10% H_2_O showed much lower sensitivity, whereas in pure methanol there was no signal at all.

**Figure 5.**
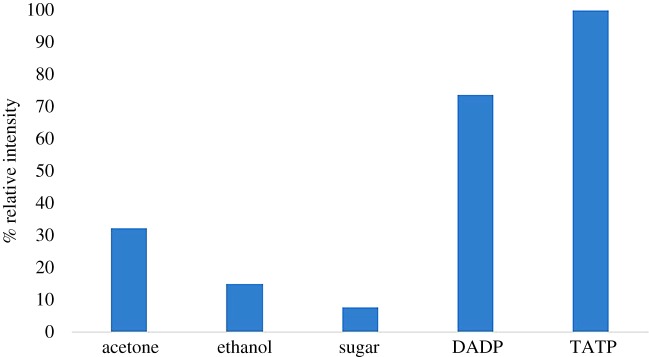
Fluorescence emission intensity changes of **1** (10^−6^ M in H_2_O : MeOH 95 : 5) in the presence of some solvents (10 µl) or sugar (0.25 mM in MeOH) compared to DADP and TATP (*λ*_exc_ = 340 nm, *λ*_em_ = 518 nm).

Finally, to study the selectivity of these systems, common solvents (acetone and ethanol) as well as commercial sugar were tested as possible interferents by fluorescence spectroscopy following the same protocol. As shown in figure 5, the response of **1** towards TATP or DADP is superior to all of them.

## 3. Conclusion

In summary, we have synthetized two new dansyl-appended β-cyclodextrin sensors and we have explored the possibility of using the well-known host–guest chemistry of cyclodextrins for the direct fluorescent sensing of the peroxide explosives DADP and TATP through a displacement approach. Host–guest experiments with β-cyclodextrin by ^1^H-NMR suggested a good binding of the DADP inside the cavity of the cyclodextrin. Although the preliminary results with sensors **1** and **2** in aqueous media show a moderate sensitivity, most probably due to solubility problems, this approach describes a new strategy for detecting peroxide explosives that has not been previously explored. Both systems showed similar fluorescent responses and both where more sensitive towards TATP than DADP.

## Supplementary Material

Supplementary Material

## Data accessibility

This article does not contain any additional data.
