# Supplementary material for: Towards the fluorogenic detection of peroxide explosives through host–guest chemistry
Source: R Soc Open Sci. 2018 Apr 11;5(4):171787. doi: 10.1098/rsos.171787 (PMC5936911; doi:10.1098/rsos.171787)
Supplement: Supplementary Material [file rsos171787supp1.pdf]

Supplementary Material

Towards the fluorogenic detection of peroxide explosives through host-guest chemistry

E. Almenar, A. M. Costero, P. Gaviña, S. Gil and M. Parra

1. General procedures

All reagents were commercially available, and were used without purification. Silica gel 60 F254 (Merck) plates were used for TLC. ^{1}H-NMR and 13C-NMR spectra were recorded on a Bruker DRX-500 spectrometer (500 MHz for 1H and 126 MHz for 13C) and a Bruker 300 MHz spectrometer. Chemical shifts are reported in ppm with the deuterated solvent as the lock and residual solvent as the internal reference. High resolution mass spectra were recorded in the positive ion mode on a VG-AutoSpec mass spectrometer. UV-vis absorption spectra were recorded using a 1 cm path length quartz cuvette on a Shimadzu UV-2101PC spectrophotometer. Fluorescence spectra were recorded using Fluoromax Horiba-MTB.

2. Syntheses

Synthesis of the peroxide explosives

TATP

In a 50 mL round bottom flask equipped with a magnetic stirring bar, 1 ml of acetone (13.5 mmol) was dissolved in 1.16 mL of hydrogen peroxide (32% v/v, 13.5 mmol). The reaction mixture was cooled to 0 ° C and 0.23 mL of conc HNO_3 was slowly added. The resulting solution was stirred at room temperature for 24 h. The resultant precipitate was isolated by filtration, washed with deionized H_2O and dried, yielding white crystals (0.52 g, 52%). The ^{1}H-NMR spectrum showed also the presence of a small amount of DADP (< 5 %)

^{1}H NMR (300 MHz, $CDCl_3$) δ 1.46 (s, 18H).

DADP[1]

Inside a 50 mL round bottom flask equipped with a magnetic stirrer 0.5 g (2.25 mmol) of TATP were dissolved in 10 mL of dry dichloromethane and a catalytic amount of p-toluenesulfonic acid was added. The reaction mixture was stirred at room temperature for one week. Then the solvent was removed under a continuous flow of argon. The solid was redissolved in CH_2Cl_2 and the mixture was washed with cold H_2O to remove the p-toluenesulfonic acid. Once isolated the solvent again removed with a continuous stream of argon to give a white solid (132 mg, 39% yield).

The ^{1}H-NMR spectrum showed also the presence of a small amount of TATP (DADP/TATP 95: 5)

^{1}H NMR (300 MHz, $CDCl_3$) δ 1.79 (s, 6H), and 1.35 (s, 6H).

Synthesis of the sensors

Mono-6-deoxy-6-tosyl-β-cyclodextrin (3).[2]

In a 250 mL round bottom flask provided with a magnetic stirrer β-CD (7.0 g, 6.2 mmol) was dissolved in 75 mL of H_2O. To the resulting whitish dispersion NaOH (2.5 g, 63 mmol) was added. The dispersion became clear gradually. After 0.5 h, 1-(p-toluenesulfonyl)imidazole (1.5 g, 6.8 mmol) was added and the reaction mixture was stirred at room temperature for 1 h. After this time the mixture was acidified with conc hydrochloric acid until pH = 5 and allowed to precipitate in the refrigerator overnight. The obtained solid was filtered under vacuum and washed with hot water, cold water and acetone respectively. Finally it was dried under vacuum to yield 2.4 g (30% yield) of a white powder.

^{1}H NMR (500 MHz, DMSO-d_6) δ 7.75 (d, *J* = 8.3 Hz, 2H), 7.43 (d, *J* = 8.1 Hz, 2H), 5.88–5.55 (m, 14H), 4.84 (m, 5H), 4.77 (m, 2H), 4.53–4.25 (m, 7H), 4.22–4.16 (m, 1H), 3.70–3.20 (m, 40H), 2.43 (s, 3H).

Mono-6-deoxy-6-azido-β-cyclodextrin (4).[3]

In a 250 mL round bottom flask equipped with a magnetic stirring bar were dissolved 0.813 g of **3** (0.63 mmol) and 0.205 g of NaN_3 (3.15 mmol) in 150 mL of H_2O and the mixture was left overnight at 86 °C. The reaction mixture was concentrated to half volume. Then 9.5 mL of 1,1,2,2-tetrachloroethane were added and the mixture was stirred for 10 min. Once the complex was formed it was separated by centrifugation (144 rpm, 10 min). The precipitate was filtered under vacuum. To remove 1,1,2,2-tetrachloroethane the mixture was dissolved in water and heated to 50 °C for 1 h. 1,1,2,2-tetrachloroethane was removed by pipette and the resulting solution was solution was dried in vacuo to yield **4** (0.4 g, 0.44% yield).

^{1}H NMR (300 MHz, DMSO-d_6) δ 5.80–5.63 (m, 14H), 4.88–4.80 (m, 7H), 4.55– 4.42 (m, 6H), 3.70–3.52 (m, 28H), 3.40–3.25 (m, 14H).

N-(but-3-yn-1-yl)-5-(dimethylamino)naphthalene-1-sulfonamide (5)

1-Amino-3-butyne (0.2 mL, 2.4 mmol) and triethylamine (0.33 mL, 2.4 mmol) were dissolved in 10 mL of dry DCM in a 50 mL round bottom flask equipped with magnetic stirrer. A solution of dansyl chloride (0.54 g, 2 mmol) in dry CH_2Cl_2 (10 mL) was added through a compensated addition funnel. The reaction mixture was left for 3 h in an ice-water bath under argon and then 3 days with vigorous stirring at room temperature. After this time the reaction mixture was washed with 35 ml of 1M NaOH and then twice with 70 mL of deionized water. It was dried with $MgSO_4$ and the solvent was removed under vacuum. The crude product was purified by column chromatography using silica gel as stationary phase and as eluent a mixture of CH_2Cl_2: MeOH 95: 5 to obtain **5** (380 mg, 63% yield).

^{1}H NMR (300 MHz, DMSO-d_6) δ 8.47 (d, *J*= 8.7 Hz, 1H), 8.27 (d, *J*= 8.7 Hz, 1H), 8.14–8.10 (m, 2H), 7.65– 7.55 (m, 2H), 7.26 (d, *J*= 7.0 Hz, 1H), 2.90 (t, *J*= 7.2 Hz, 2H), 2.83 (s, 6H), 2.77 (t, *J*= 2.7 Hz, 1H), 2.24 (dt, J= 7.2 and 2.7 Hz, 2H).

N-(pent-4-yn-1-yl)-5-(dimethylamino)-naphthalene-1-sulfonamide (6)

In a 50 mL round bottom flask equipped with magnetic bar stirring were dissolved 0.25 mL (1.8 mmol) of 4-pentyne-1-amine and 0.25 ml (1.8 mmol) of triethylamine in 10 mL dry DCM. To this solution was added, through a compensated addition funnel, 0.404 g (1.5 mmol) of dansyl chloride dissolved in 10 mL dry CH_2Cl_2. The reaction mixture was left for 3 h at 0 °C in an ice-water bath under argon and then 3 days with vigorous stirring at room temperature. After this time the reaction mixture was washed with 35 mL of 1M NaOH and then twice with 70 mL of deionized water. It was dried with $MgSO_4$ and the solvent removed under vacuum. Product purification was performed by column chromatography using silica gel as stationary phase and as eluent a mixture of CH_2Cl_2: MeOH 95: 5 to obtain 357.6 g (75% yield).

^{1}H NMR (300 MHz, $CDCl_3$) δ 8.55 (d, *J* = 8.5 Hz,1H), 8.28 (d, *J*= 9.4 Hz, 1H), 8.27–8.23 (m, 1H), 7.59-7.49 (m, 2H), 7.19 (d, J = 8.2 Hz, 1H), 4.85 (t, J = 6.3 Hz, 1H), 3.03 (q, J = 6.6 Hz, 2H), 2.89 (s, 6H), 2.12 (dt, J = 6.9 and 2.7 Hz, 2H), 1.86 (t, J = 2.7 Hz, 1H), 1.61 (t, J = 6.8 Hz, 2H)

Sensor 1

140 mg (0.46 mmol) of alkyne **5** and 640 mg (0.552 mmol) of azido-β-CD **4** were dissolved in 20 mL of DMSO in a two-necked 50 mL round bottom flask. Then 91 mg (0.46 mmol) of copper acetate were added by dragging it with 5 mL of DMSO up to a total reaction volume of 25 mL. Finally, 182 mg (0.92mmol) of sodium ascorbate dissolved in 5 mL of H_2O were added. The mixture was allowed to react for two days. After this time, the solvent mixture was removed. Once dry, it was dissolved in 30 mL of 8% aqueous NH_3 to remove the copper ion. After three days the mixture was subjected to column chromatography (MeCN:H_2O 7:3) and the fractions containing the product were combined. The solvent was removed and the crude was treated with ethyl acetate to remove excess fluorophore and finally centrifuged. The solid was washed with ethyl acetate and dried in vacuum to yield **1** (0.114 g, 17% yield).

^{1}H NMR (500 MHz, DMSO-d_6) δ 8.46 (d, *J* = 8.6 Hz, 1H), 8.28 (d, *J* = 8.7 Hz, 1H), 8.12 (d, *J* = 7.3 Hz, 1H), 7.66 (s, 1H), 7.65–7.55 (m, 2H), 7.26 (d, *J* = 7.5 Hz, 1H), 5.80–5.60 (m, 14H), 4.87–4.71 (m, 7H), 4.55–4.35 (m, 6H), 3.91 (m, 1H), 3.72-3.10 (m, 41H), 3.06 (m, 2H), 2.83 (s, 6H), 2.67 (m, 2H).
HRMS (EI): m/z calcd for $C_{58}H_{88}N_5O_{36}S$ $[M+H]^+$: 1462.493; found: 1462.489.

Sensor 2

In a 50 mL round bottom flask of two-necked 94.83 mg (0.3 mmol) of **6** and 250 mg (0.2 mmol) of **4** were dissolved in 20 mL of DMSO. Once cyclodextrin was dissolved 59.8 mg (0.3 mmol) copper acetate was added by dragging it with 5 mL of DMSO up to a total reaction volume of 25 mL. Finally, 118.86 mg (0.6 mmol) of sodium ascorbate dissolved in 5 mL of H_2O were added. The mixture was allowed to react for two days. After this time the solvent mixture was removed. Once dry, it was dissolved in 30 mL of a solution of NH_3 8% and after three days a Cu complex was formed. After this time was performed a column with MeCN:H_2O 7:3 and the fractions containing the product were combined, the solvent was removed and then redissolved in ethyl acetate to remove excess fluorophore observed in the 1H-NMR and finally centrifuged. The solid is washed with ethyl acetate and dried in vacuum obtaining 68 mg (23% yield).

[1]**H NMR** (500 MHz, DMSO-d6) δ 8.45 (d, *J* = 8.5 Hz, 1H), 8.31 (d, *J* = 8.6 Hz, 1H), 8.09 (d, *J* = 7.3 Hz, 1H), 7.70 – 7.55 (m, 2H), 7.58 (s, 1H), 7.25 (d, *J* = 7.3 Hz, 1H), 5.92–5.66 (m, 14H), 4.88–4.70 (m, 7H), 4.55–4.40 (m, 6H), 3.94 (t, 1H), 3.74 – 3.20 (m, 43 H), 2.90–2.77 (m, 8H), 1.66 (m, 2H).

HRMS (EI): m/z calcd for $C_{59}H_{89}N_5O_{36}SNa$ $[M+Na]^+$: 1498.491; found: 1498.52

3. Spectroscopic studies

All the measurements were carried out with a initial concentration of the sensor (**1** or **2**) of·10^{-6} M in H_2O:MeOH (95:5) solution. The measuring range was 400 nm to 700 nm, the excitation wavelength was 340 nm and the slits of excitation/ emission were 5/5 respectively. Both peroxide explosives were added dissolved in MeOH (5 x 10^{-4} M).

In a typical titration experiment, 2.5 mL of sensor **1** (10^{-6} M in H_2O/MeOH, 95:5) was placed inside a 1 cm path length quartz cuvette. Then 5 μL of TATP (5 x 10^{-4} M in MeOH) were added to the sensor, the mixture was heated at 40 ºC for 10 min and the fluorescence emission was recorded. The titration was performed by successive additions of 5 μL aliquots of the peroxide explosive following the same protocol. The total increase in the volume of the mixture was less than 3 %. In the preliminary experiments at room temperature, the mixture of sensor and peroxide explosive were kept 1 min at room temperature before measuring the fluorescence.

Fluorescence titration experiments at room temperature

Fig S1. Sensor 1 with TATP

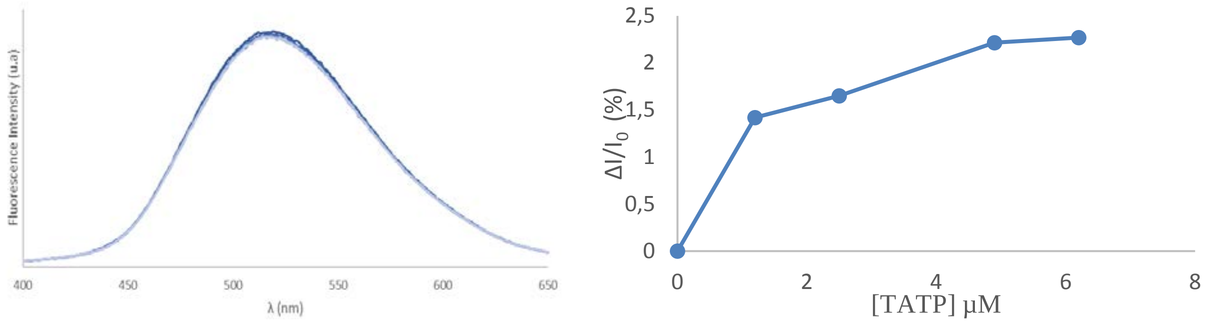

Fig S2. Sensor 1 with DADP

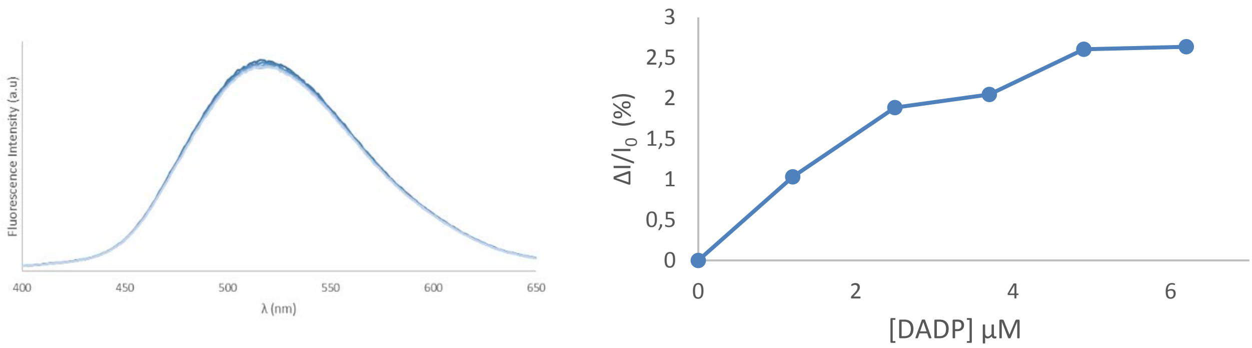

Fluorescence titration experiments with sensor 2

Fig S3. Sensor 2 with TATP

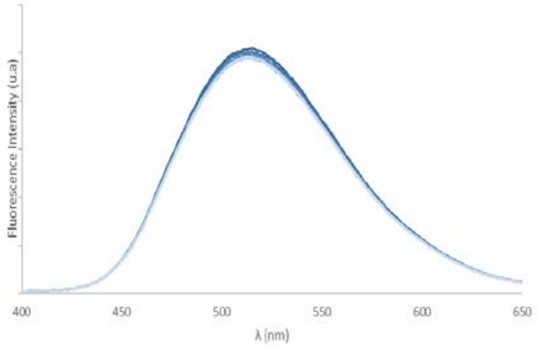

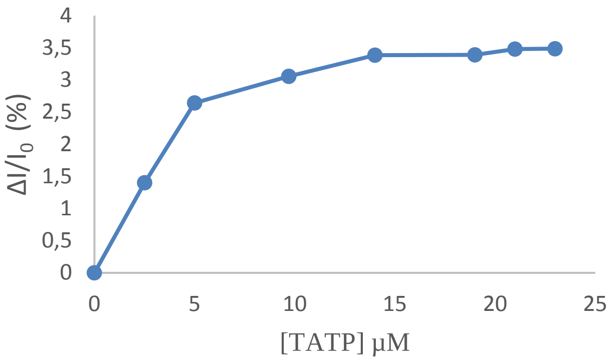

Fig S4. Sensor 2 with DADP

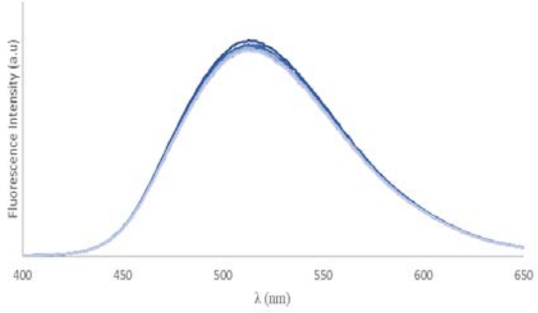

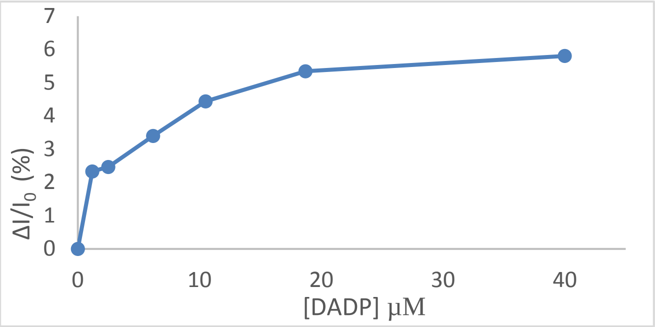

Fluorescence titration experiments with 1 after incubation at 40 ºC for 10 min

Fig S5. Sensor 1 with TATP

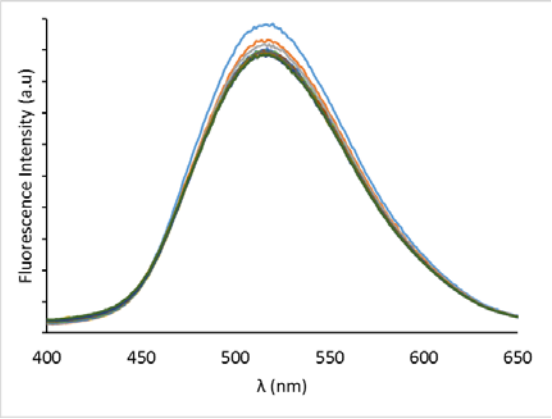

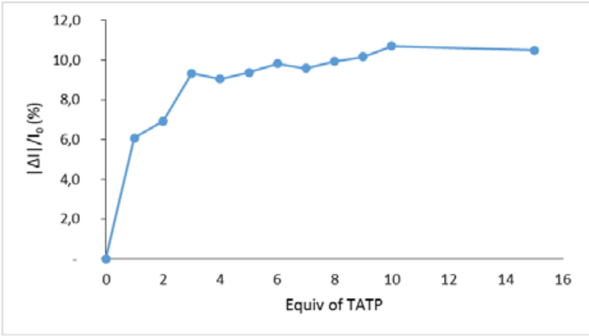

Fig S6. Sensor 1 with DADP

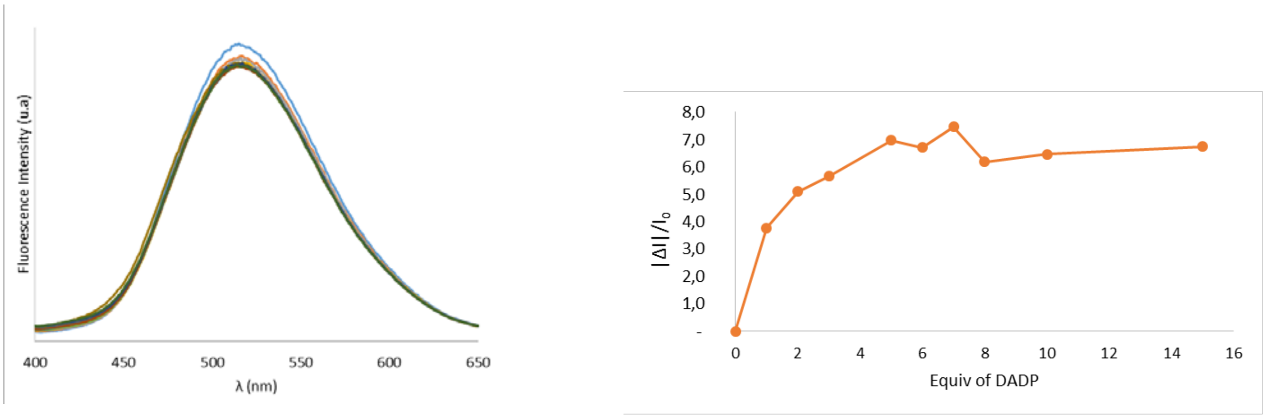

Fluorescence titration experiments with 1 and adamantane after incubation at 40 ºC for 10 min

Fluorescence titrations experiments with sensor **1** (10^{-6} M in H_2O/MeOH, 95:5) and adamantane (5 x 10^{-4} M in MeOH) were performed following the same protocol

Fig S7. Sensor 1 with adamantane

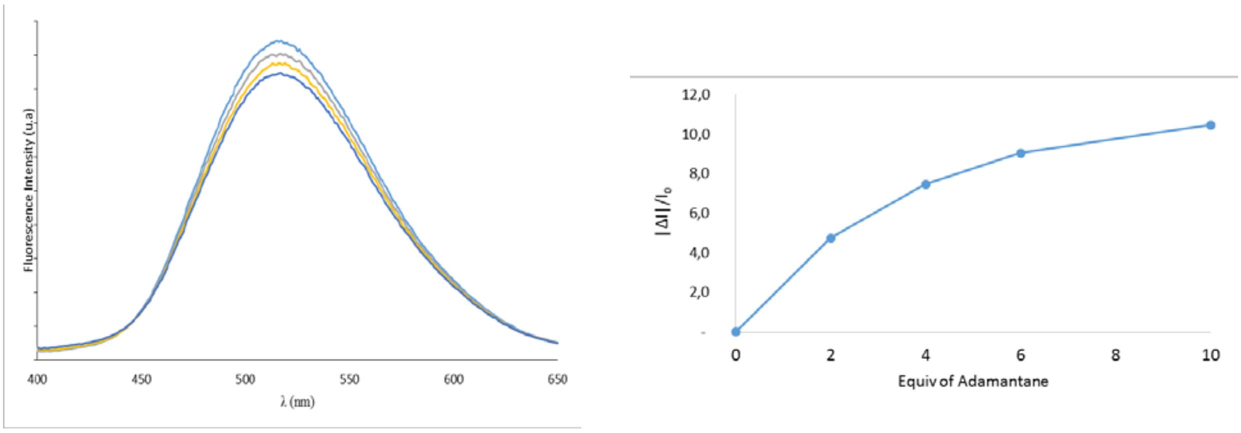

Studies with interferents

Fig S8. Sensor 1 with interferents

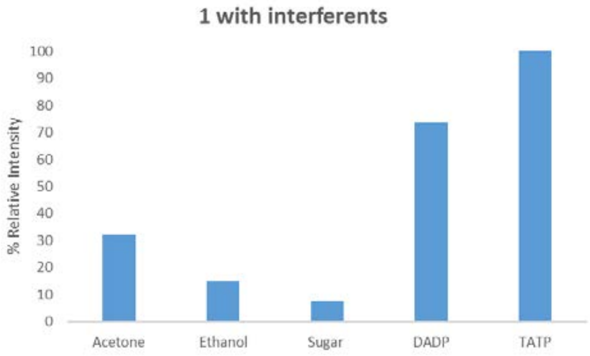

References

[1] F. Dubnikova, R. Kosloff, J. Almog, Y.Zeiri, R. Boese, H. Itzhaky; A. Alt, E. Keinan, *J. Am. Chem. Soc.* 2005, **127**, 1146.

[2] H-S. Byun, N. Zhong, R. Bittman, *Org. Synth*. 2000, **77**, 225.

[3] W. Tang, S.-C. Ng, *Nat. Protoc.* 2008, **3**, 691.
